# Therapy modifies end-vertebrae, and measuring always on the same vertebrae overestimates final results. A blind cohort controlled prospective study

**DOI:** 10.1186/1748-7161-8-S1-O30

**Published:** 2013-06-03

**Authors:** A Negrini, S Atanasio, M Vanossi, S Donzelli, F Zaina, S Negrini

**Affiliations:** 1 ISICO (Italian Scientific Spine Institute), Milan, Italy; 2 University of Brescia, Brescia, Italy; 3 IRCCS Don Gnocchi, Milan, Italy

## Background

By definition, Cobb angle is formed by the lines parallel to the superior end-plate of the superior end-vertebra (EV) and the inferior end-plate of the inferior EV, where EVs are the most tilted vertebrae. Evaluating idiopathic scoliosis (IS) results of treatment, some experts measure in different x-rays the EVs obtained at start-of-treatment (SOT); others change EVs in each single x-ray. In literature, no work ever verified if there is a difference between the two methods, nor if any agreement exists on what should be done.

## Aim

To check if there is a difference between the two ways of measurement in treated patients.

## Methods

Design is a nested cohort controlled study in a prospective database. Population Inclusion criteria: diagnosis of IS; Risser 0-3 at start; photos of x-rays at SOT, and end-of-treatment (EOT). We included 87 patients: (24) treated with Physiotherapic Specific Exercises (Group PSE: 14.7°±4.0° Cobb, 43 curves), (49) treated with Sforzesco brace+PSE (Group SF: 34.5°±8.4°, 78 curves), and (14) with SpineCor brace (Group SP: 19.6°±6.4°, 22 curves). MethodsEvery curve of each patient at SOT and EOT were measured blindly by an expert physician; EOT x-rays were blindly measured twice: on the EVs at EOT, and then on the EVs of the SOT x-ray.

## Results

EVs didn’t change between SOT and EOT in 37.2% of curves: 35.9% in PSE and SF, 45.0% in SP. The difference among groups was mainly due to distal curves (71.4% didn’t change in SP). The measurement error in this study was 0.8°±1.8° (P=0.05), while measuring always the same EVs caused an overestimation of positive results of 3.5°±2.5° (P<0.0001), up to a maximum of 18° for a curve that completely changed morphology. Considering a 5° threshold as a real difference, 9% of proximal curves and 15% of distal curves showed a change.

## Conclusion

According to our results, therapy modifies EVs. Measuring radiographs using the same EVs at SOT and EOT overestimates results. Since this study has been performed at EOT (no other changes can be expected), these results should be valid. We strongly suggest measuring the real EVs of each x-ray, particularly at EOT.
